# Cumulative Sum Analysis for Experiences of a Single-Session Retrograde Intrarenal Stone Surgery and Analysis of Predictors for Stone-Free Status

**DOI:** 10.1371/journal.pone.0084878

**Published:** 2014-01-14

**Authors:** Sung Yong Cho, Min Soo Choo, Jae Hyun Jung, Chang Wook Jeong, Sohee Oh, Seung Bae Lee, Hwancheol Son, Hyeon Jeong

**Affiliations:** 1 Department of Urology, Seoul Metropolitan Government- Seoul National University Boramae Medical Center, Seoul, Korea; 2 Department of Urology, Seoul National University Hospital, Seoul, Korea; 3 Department of Biostatistics, Seoul Metropolitan Government- Seoul National University Boramae Medical Center, Seoul, Korea; The Chinese University of Hong Kong, Hong Kong

## Abstract

**Introduction:**

This study investigated the learning curve of a single-session retrograde intrarenal surgery (RIRS) in patients with mid-sized stones. Competence and trainee proficiency for RIRS was assessed using cumulative sum analysis (CUSUM).

**Materials and Methods:**

The study design and the use of patients' information stored in the hospital database were approved by the Institutional Review Board of our institution. A retrospective review was performed for 100 patients who underwent a single-session RIRS. Patients were included if the main stone had a maximal diameter between 10 and 30 mm. The presence of a residual stone was checked on postoperative day 1 and at one-month follow-up visit. Fragmentation efficacy was calculated “removed stone volume (mm^3^) divided by operative time (min)”. CUSUM analysis was used for monitoring change in fragmentation efficacy, and we tested whether or not acceptable surgical outcomes were achieved.

**Results:**

The mean age was 54.7±14.8 years. Serum creatinine level did not change significantly. Estimated GFR and hemoglobin were within normal limits postoperatively. The CUSUM curve tended to be flat until the 25th case and showed a rising pattern but declined again until the 56th case. After that point, the fragmentation efficacy reached a plateau. The acceptable level of fragmentation efficacy was 25 ml/min. Multivariate logistic regression analyses showed that stone-free rate was significantly lower for cases with multiple stones than those with a single stone (OR = 0.147, CI 0.032 – 0.674, *P* value  = 0.005) and for cases with higher number of sites (OR = 0.676, CI 0.517 – 0.882, *P* value  = 0.004).

**Conclusions:**

The statistical analysis of RIRS learning experience revealed that 56 cases were required for reaching a plateau in the learning curve. The number of stones and the number of sites were significant predictors for stone-free status.

## Introduction

Active removal of renal stones is usually performed by extracorporeal shockwave lithotripsy (SWL), retrograde intrarenal surgery (RIRS), or percutaneous nephrolithotomy (PNL). Although many clinical parameters have affected the success rates of these treatment modalities, the main factor for determination of stone removal method has been the stone size [1], [2]. SWL showed excellent stone-free rates (SFRs) for stones up to 20 mm, and PNL has been the primary treatment method for large stones with maximal diameter >20 mm [3]. Previous studies on SWL have identified unfavorable factors for SFR such as stones in the lower-pole calyx, steep infundibular-pelvic angle, long calyx, narrow infundibulum, hard stones, or obesity, which often required multiple treatments or ancillary procedures [4], [5]. Although PNL was associated with high postoperative SFR, this surgical procedure is associated with disadvantages, such as risk of renal damage, bleeding, or perioperative hemoglobin drop, because this technique should penetrate the renal parenchyme [6].

RIRS has shown a high SFR comparable to that of PNL, and offers the possibility of less renal injury than PNL because this procedure does not penetrate the renal cortex [7]. Furthermore, RIRS can address the limitations associated with SWL such as lower-pole calyx stones, obesity, etc. However, the advantages of RIRS should not be overemphasized because of disadvantages such as short-lived durability of the flexible ureteroscopic equipment [8], possibility of staged procedures, or ureteral injury [9]. Few studies have reported cases in which RIRS was able to retrieve stones up to 3 cm in size, depending on the skill level of the operating surgeon [3], [10]. Considering these factors, RIRS appears to be a good option for mid-sized stones with diameters between 10 and 30 mm at high-volume centers. In order to adopt RIRS, endeavors to overcome the surgical difficulties would be important, especially for the less-experienced trainees in the urologic field; however, few investigations have shown the learning curve to acquire the appropriate surgical proficiency for RIRS.

Therefore, the present study investigated the learning curve of a single-session RIRS in patients with a main stone larger than 10 mm. Competence and trainee proficiency for RIRS was assessed using cumulative sum analysis (CUSUM). The predictors for stone-free status were evaluated.

## Materials and Methods

### Subjects

The study design and the use of patients' information stored in the hospital database were approved by the Institutional Review Board (IRB) at the Seoul Metropolitan Government - Seoul National University Boramae Medical Center. The approval number is 16-2012-21. We were given exemption from getting informed consents by the IRB because the present study is a retrospective study and personal identifiers were completely removed and the data were analyzed anonymously. A retrospective review was performed for 100 consecutive patients who underwent a single-session RIRS at a single center from 2011 to 2012. Patients were included in the analysis if the main stone had a maximal diameter between 10 and 30 mm. Patients with febrile urinary tract infection, bleeding tendency, anatomical anomaly, or ureteral stricture were excluded from the analysis. Patients with bilateral stones were also excluded in the analysis.

### Surgical methods

All patients underwent cystoscopic examinations in the dorsal lithotomy position. A 0.035-mm Terumo guidewire (Boston Scientific Corporation, Miami, FL, USA) was inserted into the ureteral orifice. The Terumo guidewire was exchanged with a Superstiff guidewire (Boston Scientific Corporation, Miami, FL, USA) using an open-ended 5-Fr ureteral catheter. When there was a definite evidence of ureteral stricture, the ureteral JJ stent inserted preoperatively was retracted and a Superstiff guidewire was inserted into the ureteral JJ stent. After a 14- or 12-Fr ureteral access sheath (Cook Medical, Bloomington, IN, USA) was inserted into the level of ureteropelvic junction, a 7.5-Fr flexible ureteroscope (Flex-X^2^TM, Karl Storz, Tuttlingen, Germany) was inserted through the access sheath. If a 12-Fr access sheath could not be inserted due to the ureteral narrowing, the following maneuvers were performed: (i) the Superstiff guidewire was changed again into the Terumo guidewire, which was inserted into the working channel of the distal tip. The flexible ureteroscope was inserted into the renal pelvis through the Terumo guidewire; (ii) a ureteral balloon dilatation was performed if the ureter was too narrow for the flexible ureteroscope to be inserted; (iii) a 6-Fr nelaton catheter was inserted into the bladder through the urethra to prevent bladder filling which might contribute to the compression of ureterovesical junction, increase in the intrarenal pressure, and limit of the motion of flexible ureteroscope.

After the distal end of the scope was placed in the renal pelvis, a 365- or 200-µm laser fiber was used for stone fragmentation. Stones which were located in the deep area of renal collecting system were fragmented by 200-µm laser fiber to acquire maximum deflection of the distal ureteroscopic tip, or the stones were mobilized into the upper or middle calyceal space before fragmentation. Holmium laser power was set to 10 W. The repetition rates were 15 Hz and 7 Hz for 365- and 200-µm laser fibers, respectively. An endoscopy irrigation pump (Stryker, Kalamazoo, MI, USA) was used to maintain a constant intrarenal pressure of 50 to 120 mmHg. A 1.9-Fr zero-tipped nitinol stone basket (Cook Medical) was usually used for stone removal after fragmentation. Additionally, the laser was fired in a continuous stream, which was focused on the surface of the renal stone until it broke into fragments less than 1 mm. A 6-Fr JJ ureteral stent was routinely placed to provide a conduit for the fragmented stones to enter the bladder. A urethral Foley catheter was placed into the bladder postoperatively. Most patients were discharged on the first postoperative day after the urethral Foley catheter was removed. The ureteral JJ stent was usually removed 1 to 2 weeks postoperatively.

### Clinical parameters

All patient data were reviewed for age at operation, gender, height, body weight, serum creatinine, estimated GFR, and hemoglobin. For each patient, the stone parameters evaluated were the number of stones, stone location, major stone composition, previous treatments for stone, stone diameters, average Hounsfield units, and stone volume (length × width × height × 0.523 mm^3^). Total stone volume was the sum of each stone volume. All patients underwent computed tomography (CT) preoperatively. The operative time was defined as the time that passed from insertion of an endoscope into the urethra to the completion of urethral stent placement. On postoperative day 1 and at the one-month follow-up visit, patients underwent X-ray of the KUB (kidney, ureter, and bladder) or non-contrast CT to evaluate for the presence of residual stone. One of these two imaging modalities was chosen depending on the radiopacity of the stone. The average Hounsfield unit was evaluated by an ellipsoid region of interest on the axial images of CT. A staghorn stone was defined as a stone located in the renal pelvis that extended into more than one calyx. Stone location was classified as renal pelvis, superior/inferior major calyces, and anterior/posterior minor calyces of the superior/middle/inferior calyces. The anterior and posterior divisions were determined by using the extended frontal plane of the major calyx [11]. The presence of stone was checked in each site. Intra- and perioperative parameters were also evaluated. Clinically “stone-free” status was defined as no evidence of stones or stones less than 1 mm on one-month postoperative images. Complications were assessed according to the modified Clavien classification. Fragmentation efficacy was calculated “removed stone volume (mm^3^) divided by operative time (min)”, and was evaluated in the sequential order of operations.

### Statistical analyses

All parameters were represented as mean ± standard deviation or frequency (percentage). Patient demographics were analyzed using independent t-test or Mann-Whitney U test between the two groups. The Chi-square and Fisher's exact test were used for analysis of categorical variables. Data for the learning curve evaluations were analyzed by the independent t-test, analysis of variance (ANOVA), and chi-square tests. Post-hoc analysis was performed by Tukey method.

CUSUM analysis was used for monitoring change in fragmentation efficacy, and we tested whether or not acceptable surgical outcomes were achieved [12]. CUSUM analysis is a graphical method of quality control that examines consecutive series of procedures to determine trends in changes over time [13]. To quickly detect an acceptable level of performance, CUSUM chart is displayed with CUSUM values on the *y*-axis against the series of procedures on the *x*-axis. The CUSUM value for *n*th procedure is defined as *S_n_*  =  *max*(0, *S_n_*_-1_ + *X_n_* – *c*), where *S*_0_ is 0 at the start, *X_n_* is the standardized value for *n*th procedure, and *c* is the reference value and defined by pre-specified standard of performance for the procedure to be monitored. Since CUSUM function is the maximum function, it applies to monitoring to identify an acceptable level of performance (upward CUSUM) [12]. The CUSUM curve usually runs randomly without any slope; however, a trainee acquiring a new skill in any surgical procedure would be expected to have a rising CUSUM curve. When a trainee masters the skill, the curve eventually flattens without any slope. Additionally, a change point analysis was also performed to identify the point at which the statistical properties of a sequence of observation changes in mean and/or variance under distribution-free circumstances [14]. We focused on the detection of the single change point in mean.

Linear regression analysis was used to evaluate the relationship between fragmentation efficacy and either the stone size or the volume. Univariate and multivariate logistic regression analyses with backward stepwise selection were used to show predictors for stone-free status. Statistical significance was considered at *P*<0.05. Statistical analyses were performed by IBM SPSS Statistics version 20 (IBM Inc., Chicago, IL, USA) and R version 3.0.1 (http://www.r-project.org).

## Results

### Patient characteristics

As shown in Table 1, the mean age was 54.7±14.8 years. Serum creatinine level did not change significantly. There were significant changes in estimated GFR and hemoglobin drop perioperatively; however, all postoperative findings were within normal limits.

**Table 1:** Patients and stone characteristics.

				Mean ± SD or N (%)
N				100 (100%)
**Patient characteristics**				
Age, year				54.7±14.8
Gender				
			Male	62 (62.0%)
			Female	38 (38.0%)
Body mass index, kg/m^2^				24.8±3.9
Creatinine, mg/dl				
			Preoperative	1.02±0.5
			postoperative	1.04±0.2
			P value	0.558
Estimated GFR, mL/min/1.73 m^2^				
			Preoperative	79.2±29.3
			GFR increase (post- minus preoperative)	5.2±19.2
			P value	**0.044** *
Hemoglobin, mg/dl				
			Preoperative	13.4±1.9
			Hemoglobin drop (post- minus preoperative)	−0.9±1.3
			P value	**<0.001** *
**Stone characteristics**				
Previous SWL history (+)				16 (16.0%)
Previous URS history (+)				25 (25.0%)
Stone site				
		Right		54 (54.0%)
		Left		46 (46.0%)
Stone location				
		Pelvis		90 (90.0%)
		Major calyx (superior)		14 (14.0%)
		Major calyx (inferior)		16 (16.0%)
		Minor calyx (superior anterior)		12 (12.0%)
		Minor calyx (superior posterior)		28 (28.0%)
		Minor calyx (middle anterior)		14 (14.0%)
		Minor calyx (middle posterior)		19 (19.0%)
		Minor calyx (inferior anterior)		37 (37.0%)
		Minor calyx (inferior posterior)		32 (32.0%)
Hounsfield unit				822.0±362.2
Main stone composition				
	Calcium oxalate monohydrate			19 (19.0%)
	Uric acid			9 (9.0%)
	Carbonate apatite			6 (6.0%)
	Calcium oxalate dehydrate			1 (1.0%)
	Struvite			1 (1.0%)
	Others			5 (5.0%)
	Missing			59 (59.0%)

P value <0.05.

### Surgical outcomes in the order of sequential cases

A single surgeon (Cho S.Y.) performed the 100 consecutive cases without any significant mechanical failure from the single flexible Flex-X^2^™ ureteroscope. There were several events in which the sheath was perforated during the insertion of the laser fiber, and each of these holes was repaired within postoperative 2 to 3 days. Table 2 shows the changes in perioperative parameters by the sequential order of cases. Although operative times increased significantly after the first 60 cases, these increases corresponded with significant increases in maximal stone sizes, total stone volumes, and fragmentation efficacy. When surgical outcomes were analyzed in sets of 10 cases, there was no significant difference in fragmentation efficacy between the cases 51 to 60 and the next 10 cases in post-hoc analysis. Therefore, in terms of fragmentation efficacy, 50 cases are required for reaching the plateau of a learning curve. Although there were significant differences in operative parameters, no significant difference in stone-free status was identified between the initial 10 and the final 10 cases. Clinically stone-free status was 79% and 92% for no stones and stones less than 2 mm, respectively. Postoperative complications had occurred in 6 cases, 1 case of fever (Clavien classification grade I), 2 cases of acute pyelonephritis (grade II), and 1 case of bleeding without embolization (grade II). There was no complication of sepsis or ureteral stricture during the follow-up period from 6 to 12 months. All complications were mild and were managed properly.

**Table 2:** Perioperative findings and surgical outcomes according to the number of cases.

	Total	0–20	21–40	41–60	61–80	81–100	P value
**Operative parameters**							
Operative time, min	98.0±78.7	105.0±61.9	86.7±54.8	55.6±54.3	129.4±113.2†	113.2±104.6†	**0.032** *
Maximal stone size, mm	19.2±11.5	15.3±4.4	18.8±10.8	16.1±9.8	25.9±17.2†	19.9±9.4	**0.028** *
Total stone volume, mm^3^	3265.6±5591.8	1162.3±989.7	1905.3±2813.1	2408.2±6471.3	5472.0±7458.2†	5380.0±6270.3†	**0.030** *
Number of stones	5.7±3.8	2.0±1.4	2.5±1.3	2.5±2.1	3.2±3.4	2.4±1.5	0.483
Fragmentation efficacy, ml/min	26.8±25.6	12.3±9.6	20.2±19.5	22.8±24.5	35.9±32.0†	42.9±25.9†	**<0.001** *
Estimated blood loss, ml	10.3±20.2	10.3±14.0	8.8±16.8	5.0±0.0	22.5±37.9†	5.0±0.0	**0.035** *
Removal of ureteral JJ stent, day	11.1±3.5	10.0±0.0	10.6±2.5	11.7±4.0	11.7±4.0	11.5±4.9	0.461
Removal of urethral catheter, day	1.4±1.2	1.3±0.7	1.6±1.7	1.0±0.3	1.4±1.2	1.7±1.7	0.429
Discharge, day	2.0±2.3	3.0±3.7	2.4±2.6	1.2±0.7	1.6±1.4	1.9±1.7	0.102
**Surgical outcomes**							
No evidence of stone	79 (79.0%)	14 (70.0%)	14 (70.0%)	18 (90.0%)	17 (85.0%)	16 (80.0%)	0.426
Stone <1 mm	92 (92.0%)	18 (90.0%)	18 (90.0%)	19 (95.0%)	18 (90.0%)	19 (95.0%)	0.936
Complications	6 (6.0%)	1 (5.0%)	0 (0.0%)	1 (5.0%)	4 (20.0%)†	0 (0.0%)	0.048

P value <0.05, Mean ± SD or N (%).

Significantly higher than the other parameters without the mark by post-hoc analysis.

### CUSUM analysis

**Figure 1:**
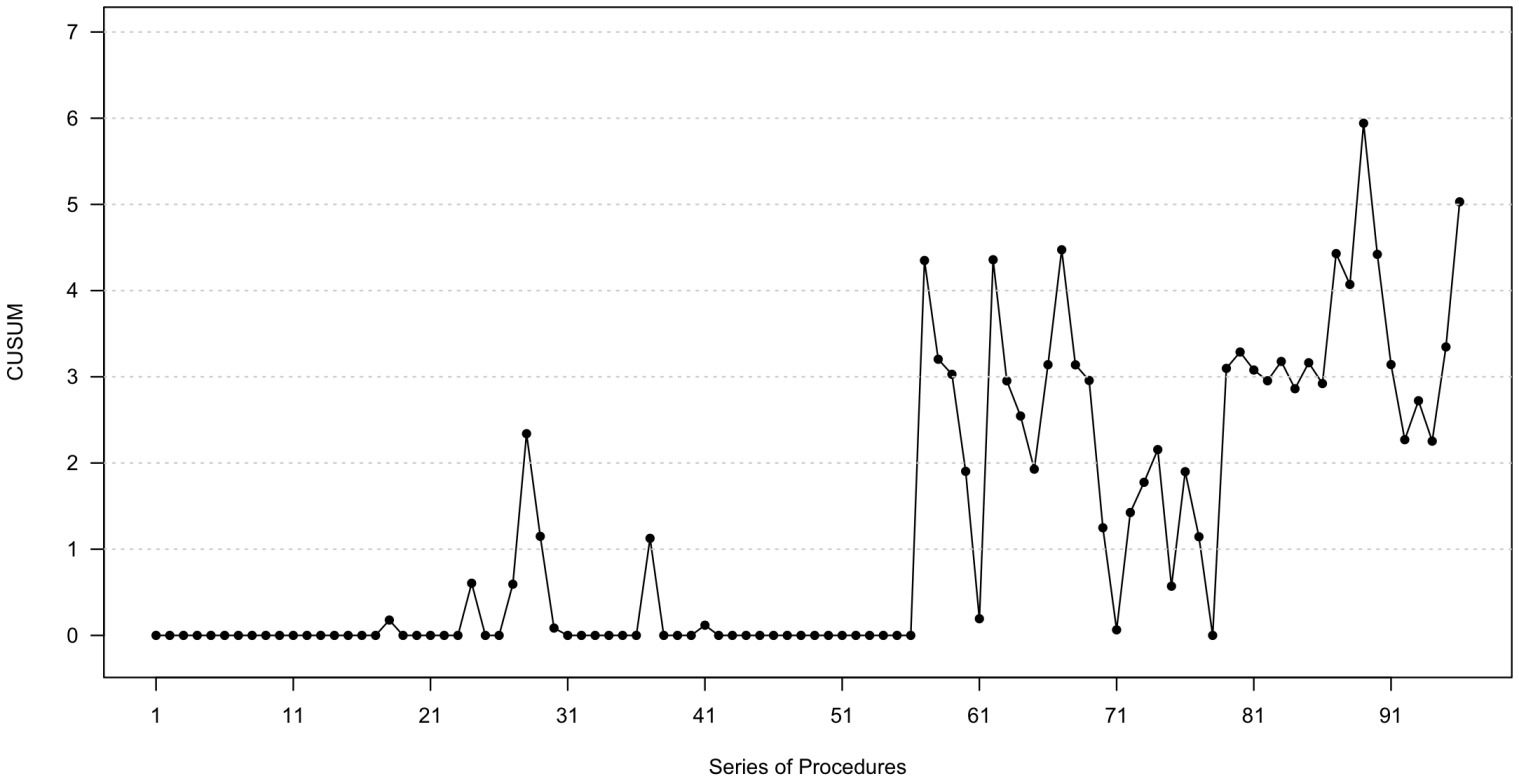
The upward CUSUM chart. The CUSUM curve tended to be flat until the 25th case. After the initial 25 cases, the curve showed a rising pattern but declined again. However, CUSUM curve showed an upward shift. After the 56th case, the change point analysis result presented the 56th case was the mean change point. As a result, the fragmentation efficacy reached a plateau from this point.

Figure 1 shows the upward CUSUM chart. During the trial-and-error period, the CUSUM curve tended to be flat until the 25th case. After the initial 25 cases, the CUSUM curve showed a rising pattern but declined again until the 56th case. After that point, CUSUM values showed an upward shift, which meant that the acceptable level of performance occurred. Thus, the fragmentation efficacy reached a plateau. The change point analysis result also indicated that the 56th case was the mean change point. The acceptable level of fragmentation efficacy was a minimum of 25 ml/min, which is equal to a one-hour surgery for a calculated stone volume of 20 mm×15 mm×10 mm×0.523.

### Analysis for prediction of stone-free status

Univariate and multivariate logistic regression analyses showed that significant predictors for stone-free status were the number of stones (1 vs ≥2) and the number of sites involved as shown in Table 3.[11] Stone-free rate was significantly lower for cases with multiple stones than those with a single stone (OR = 0.147, CI 0.032 – 0.674, *P* value  = 0.005) and for cases with higher number of sites (OR = 0.676, CI 0.517 – 0.882, *P* value  = 0.004). Using the results of logistic regression analyses, differences in parameters of the number of stones (1 vs ≥2) and the number of sites involved were further evaluated. When patients of a single stone were compared to those with multiple stones, there were no differences in clinical parameters except for the number of sites involved (1.4 vs 3.3, *P* value <0.001). Comparing the number of sites between 5 and 9 and the number of sites less than 5, maximal size (34.6±21.0 vs 16.7±6.4 mm, *P* value <0.001), total number of stones (4.6±3.6 vs 2.2±1.5, *P* value  = 0.027), total stone volumes (11852.2±10435.3 vs 1867.7±2375.4 mm^3^, *P* value  = 0.003), operative times (212.6±113.8 vs 79.3±52.1 m, *P* value  = 0.001), fragmentation efficacy (53.1±36.8 vs 22.5±20.5 mm^3^/min, *P* value  = 0.009), and stone-free rates (42.9% vs 84.9%, *P* value <0.001) were significantly different between the two groups.

**Table 3:** Uni- and multivariate logistic regression analysis to predict clinically stone-free status.

	Univariate			Multivariate		
	P value	OR	95% CI	P value	OR	95% CI
Age	0.508	0.983	0.933 – 1.035			
Gender (male versus female)	0.470	0.586	0.138 – 2.497			
Body mass index	0.615	0.952	0.786 – 1.153			
Preoperative SWL history	0.629	0.576	0.062 – 5.375			
Preoperative URS history	0.999	-	-			
Laterality (left versus right)	0.049*	0.301	0.091 – 0.996			
Number of stones (1 or ≥2)	0.018*	0.069	0.007 – 0.636	0.018*	0.118	0.020 – 0.691
Maximal stone size	0.369	1.049	0.945 – 1.163			
Total stone volume	0.313	1.000	1.000 – 1.000			
Hounsfield unit	0.345	0.999	0.997 – 1.001			
Number of sites involved	0.041*	0.678	0.413 – 1.113	0.006*	0.124	0.028 – 0.547
Use of access sheath	0.906	0.917	0.216 – 3.888			
Fragmentation efficacy	0.846	1.004	0.967 – 1.042			

PNL: percutaneous nephrolithotomy, RIRS: retrograde intrarenal stone surgery, SWL: extracorporeal shock wave lithotripsy, URS: ureteroscopic stone surgery.

When clinical parameters were compared between patients with residual stones and those without residual stones, total number of stones (3.5±3.1 vs 2.2±1.6, *P* value  = 0.011), total stone volume (6888.9±9638.1 vs 2302.4±3375.3 mm^3^, *P* value  = 0.044), operative time (149.2±95.5 vs 84.3±68.0, *P* value  = 0.001), and the number of sites involved (3.9±2.5 vs 2.3±1.6, *P* value  = 0.010) were significantly different. Other parameters including age, gender, body mass index, serum creatinine level, maximal stone size, and fragmentation efficacy showed no significant differences.

## Comment

### RIRS and a new option and learning curve

After introduction of RIRS, urologists usually have removed renal stones by using SWL, PNL, and RIRS. Although previous studies demonstrated the advantages and high success rates of PNL in the management of large renal stones, experiences with RIRS have revealed comparable SFR with less risk of renal damage and bleeding [7]. Recently, some investigations demonstrated that RIRS can be a good management option for mid-sized stones between 1 to 4 cm [3], [10]. However, a steep learning curve and short-lived durability of flexible ureteroscopic equipment have disappointed surgeons who are uninitiated with the RIRS techniques [8].

Although many surgeons have reported outcomes from using flexible ureteroscopes, there has been no report on the learning curve of RIRS. These surgeons performed RIRS for management of renal stones of all sizes from less than 1 cm to 4 cm [3], [10], and the surgical techniques differed a great deal from report to report. Recently, the guidelines of European Association of Urology reported that RIRS can be a good management option for mid-sized stones from 1 to 3 cm at high-volume centers, and some investigations have shown increasing durability of flexible ureteroscopes [15]. In the present study, the surgeon (Cho S.Y.) had performed 100 consecutive cases without any significant mechanical failure of the single flexible Flex-X^2^™ ureteroscope. Therefore, the authors believe that this was an appropriate time to report on the learning curve of RIRS. Because there was no proper instructor, the present study would be greatly helpful for surgeons who would have to learn to perform RIRS by themselves.

### Acceptable level of fragmentation efficacy

As previously mentioned, there has been no report of learning curve of RIRS, and understandably, no study has investigated the appropriate objective parameter to evaluate competency. The authors have suggested the fragmentation efficacy as a good parameter to evaluate the competency of RIRS. This efficacy was calculated by the formula of ‘removed stone volume (mm^3^) divided by operative time (min)’, which answers the clinical question “How fast can a given surgeon perform RIRS?” This idea is similar to the one behind enucleation efficacy of Holmium laser enucleation for prostate [16]. As shown in Table 2, maximal stone size and stone volume increased according to the number of cases; however, the fragmentation efficacy reached a plateau in the interval from the 40^th^ to the 60^th^ case. For demonstrating the quality of procedure, we used CUSUM analysis to identify and evaluate the change point and the presence of a plateau. The CUSUM curve indicated the change point at the 56^th^ case, and the minimally acceptable level of fragmentation efficacy was 25.0 ml/min, which means one-hour surgery for a stone with dimensions measuring 20 mm×15 mm×10 mm. This is not an absolute goal of beginners and rather indicates an effectiveness of operations, which can meet a minimally agreed standard. Previous studies showed similar values of stone surface area or stone burden [7], [17]. They were not appropriate for beginners to accept as the appropriate acceptable level of performance, because it was not obscure that the cases were during the surgeons' learning curve. The appropriate standard can be elevated in the near future as the surgeons overcome surgical difficulties of RIRS; however, the authors think that 25.0 ml/min in the present study would be appropriate for beginners to accept as the minimally acceptable level of performance.

### Technical challenge of RIRS

Previous studies reported increasing durability of flexible equipments, and recent investigations have shown that more than 100 procedures could be performed with one or two ureteroscopes with minimal damage to the equipment, depending on the level of care the instruments received [8], [18]. The surgeon in the present study performed all of the cases without any significant mechanical failure of the single flexible ureteroscope (Flex-X^2^™), except for the perforations in the distal tip sheath. The sheath was perforated by the laser fiber, and this was repaired within 2-to-3 days if the hole was detected immediate postoperatively. Surgeons should follow the user guidelines for flexible ureteroscopes [8], and try to expand longevity of flexible ureteroscopes as much as possible.

### Limitation of this study

One of the main limitations of the present study is the retrospective nature of the study. The fragmentation efficacy is calculated by operative time (min), which is different from time duration for laser. We could neither measure laser duration in all procedures nor compare the fragmentation efficacies calculated by the operative time and the laser duration. Further study can compare these different estimation methods of surgical efficiency. Another limitation is inaccurate calculation of stone volume because we could not collect all stones which were fragmented by laser until they became dust. The stone is hypothesized to be oval in shape and stone volume was calculated by the formula of length × width × height ×0.523 mm^3^. Lastly, the results of the present study are based on the learning experience of a single surgeon and require external validation.

## Conclusions

The statistical analysis of RIRS learning experience revealed that 56 cases were required for reaching a plateau in the learning curve. The number of stones and the number of sites were significant predictors for stone-free status.
